# Comparison of segmental spinal movement control in adolescents with and without idiopathic scoliosis using modified pressure biofeedback unit

**DOI:** 10.1371/journal.pone.0181915

**Published:** 2017-07-28

**Authors:** Hong-Ji Luo, Shi-Xiang Lin, Shyi-Kuen Wu, Mei-Wun Tsai, Shwn-Jen Lee

**Affiliations:** 1 Department of Physical Therapy and Assistive Technology, National Yang-Ming University, Taipei, Taiwan; 2 Research Center on International Classification of Functioning, Disability and Health (ICF) and Assistive Technology, National Yang-Ming University, Taipei, Taiwan; 3 Department of Physical Therapy, Hungkuang University, Taichung, Taiwan; University of Illinois at Urbana-Champaign, UNITED STATES

## Abstract

**Background:**

Postural rehabilitation emphasizing on motor control training of segmental spinal movements has been proposed to effectively reduce the scoliotic spinal deformities in adolescent idiopathic scoliosis (AIS). However, information regarding the impairments of segmental spinal movement control involving segmental spinal stabilizers in adolescent idiopathic scoliosis remains limited. Examination of segmental spinal movement control may provide a window for investigating the features of impaired movement control specific to spinal segments that may assist in the development of physiotherapeutic management of AIS.

**Objectives:**

To compare segmental spinal movement control in adolescents with and without idiopathic scoliosis using modified pressure biofeedback unit.

**Methods:**

Segmental spinal movement control was assessed in twenty adolescents with idiopathic scoliosis (AISG) and twenty healthy adolescents (CG) using a modified pressure biofeedback unit. Participants performed segmental spinal movements that primarily involved segmental spinal stabilizing muscles with graded and sustained muscle contraction against/off a pressure cuff from baseline to target pressures and then maintained for 1 min. Pressure data during the 1-minute maintenance phase were collected for further analysis. Pressure deviation were calculated and compared between groups.

**Results:**

The AISG had significantly greater pressure deviations for all segmental spinal movements of cervical, thoracic, and lumbar spine than the CG.

**Conclusion:**

Pressure biofeedback unit was feasible for assessing segmental spinal movement control in AIS. AISG exhibited poorer ability to grade and sustain muscle activities for local movements of cervical, thoracic, and lumbar spine, suggesting motor control training of segmental spinal movements involving segmental spinal stabilizing muscles on frontal, sagittal, and transverse planes were required.

## Introduction

Adolescent idiopathic scoliosis (AIS), developing at the ages of 11–18 years, is a deformity of the spine and trunk involving all three planes with spinal curvature greater than 10 degrees measured using Cobb method [1] and accounting for approximately 90% of idiopathic scoliosis in children [2]. On inspection, AIS is generally characterized by asymmetric, lateral-shift, and curved trunk accompanying rib and back hump and axial twist of torso that tend to deteriorate rapidly during the very fast-growing period [3, 4] and adversely influence physical and psychological health as well as quality of life of AIS [5, 6]. Although the definite etiology of AIS remains unclear, genetic expression along with environmental and lifestyle factors [7] may be responsible for impairments at neuro-osseous [8], biomechanical (e.g., asymmetrical vertebrae growth) [9], neuromuscular (e.g., disorganized sensorimotor integration or sensory processing) [7, 10], and cellular (e.g., melatonin signaling malfunction) [7, 11] levels in AIS.

In terms of neuromuscular perspective, impaired motor control of the axial motor system may be the cause of AIS [12, 13]. Studies indicated that AIS assumes altered motor strategy based on re-weighting of proprioceptive inputs arising from axial musculature to restore perceptual-motor dysfunction [14, 15]. In addition, electromyography study of the activities of paraspinal muscles revealed that AIS demonstrated asymmetric firing of bilateral paraspinal muscles with higher muscle activities on the convex side than the concave side [16, 17]. Furthermore, individuals with AIS demonstrate asymmetric muscle thickness of the deep thoracic paraspinal muscles [18], lumbar multifidus [18, 19], obliquus internus and externus [20], and transverse abdominus [21] on the concave and convex sides. These muscles play important roles on the regulating segmental spinal alignment and stability [22–24] to ensure a stable base for force transmission through kinetic chains and efficient functional performance. However, morphological imbalance and neurophysiological dysfunction in these muscles may negatively affect the segmental spinal stability and maintenance of neutral spinal alignment in AIS during static and dynamic activities. Therefore, examination of segmental spinal movement control may provide crucial information to inform physiotherapists in designing exercise therapy programs for AIS.

The movements specifically to local spinal segments in individuals with AIS have been seldom examined previously. Shirado et al. [25] examined dynamic lateral side-shift movements of trunk in sitting and found that individuals with AIS showed impaired trunk movement control by revealing a lesser shifted weight and a longer time to shift weight than those without AIS at both slow and fast speeds. Guyot et al. [26] examined local movement of cervical spine and reported that individuals with AIS performed poorly on the cervicocephalic relocation test compared with healthy individuals without AIS, suggesting a dysfunctional control of segmental cervical spine movement in anterior-posterior direction (sagittal plane) while primarily activating cervical stabilizing muscles (i.e., deep cervical flexor muscles). However, in AIS, information regarding segmental spinal movements primarily involving local spinal stabilizing muscles around thoracic and lumbar regions and in all three planes remains limited. Therefore, movements of local spinal segments in individuals with AIS must be investigated in order to assist researchers and clinicians in characterizing deviated motor performance of local spinal segments in AIS, thus providing further insight into the specific physiotherapeutic strategies for managing AIS.

In both research and clinical settings, a pressure biofeedback unit has been used to assess and train lumbar–pelvic movement control primarily by activating segmental spinal stabilizing muscles in individuals with lower back pain [27–29] as well as cervical dysfunction [29, 30]. The way of using pressure biofeedback units could be also suitable for assessing segmental spinal movements in AIS. Therefore, this study aimed to examine segmental spinal movements primarily by activating local spinal stabilizing muscles in adolescents with and without idiopathic scoliosis using modified pressure biofeedback unit.

## Materials and methods

An exploratory cross-sectional study design was used. Adolescents with idiopathic scoliosis (AISG) and healthy adolescents without idiopathic scoliosis (CG) were included and assessed for their segmental spinal movement control once by using a modified pressure biofeedback unit. This study was conducted at Child Development Laboratory of National Yang-Ming University in accordance with the Declaration of Helsinki. This research protocol was reviewed and approved by the Institutional Review Board of National Yang-Ming University (YM105010F). Informed consent was received from both participants and their parents before recruitment in this study.

### Participants

All participants were recruited from primary and high schools in Northern Taiwan. The common inclusion criteria for both the AISG and CG are outlined as follows: (1) age of 10–18 years, (2) medical clearance for other disorders that might affect truncal and spinal movements, and (3) ability to follow instructions to perform testing movements. The exclusion criteria for both groups are outlined as follows: (1) any numbness, weakness, or paresthesia in the extremities; (2) a history of surgery of the trunk, spine, or lower extremities; and (3) leg length discrepancy (>1 cm). Furthermore, the participants in the AISG were required to have a confirmed diagnosis of AIS with a Cobb angle of 10°–40°.

### Segmental spinal movement assessment

**Fig 1:**
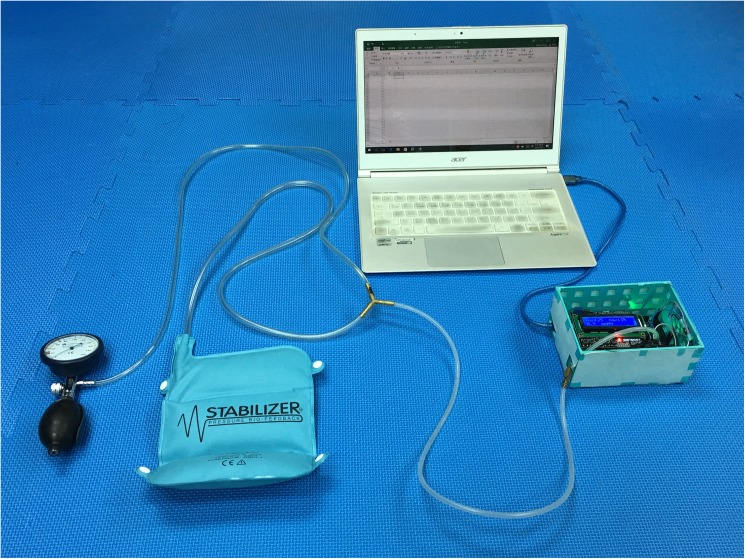
Modified pressure biofeedback unit.

A three-way adapter connecting the air pump and pressure cuff of a commercially available pressure biofeedback unit (Stabilizer™ Pressure Biofeedback, Chattanooga Group, Australia) and a pressure sensor (range: 0–75 mmHg; accuracy: 0.1 mmHg) (MPX5010DP, Freescale Semiconductor Inc., USA) that was mounted on a microcontroller board (Arduino Compatible Mega 2560 R3, Arduino LLC., Italy) constituted the modified pressure biofeedback unit used in this study (Fig 1). Check for the leak of air from the adapter was performed under water and no leak of air was found.

**Fig 2:**
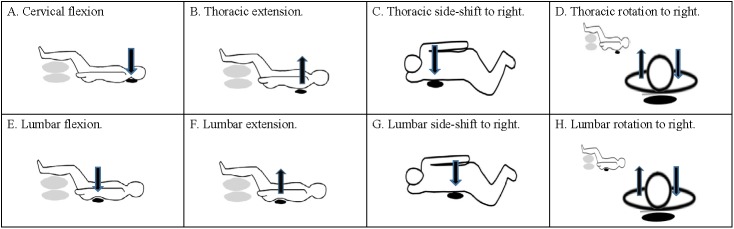
Illustrations of test setting. (A) Cervical flexion. (B) Thoracic extension. (C) Thoracic side-shift to right. (D) Thoracic rotation to right. (E) Lumbar flexion. (F) Lumbar extension. (G) Lumbar side-shift to right. (H) Lumbar rotation to right. Black oval denotes pressure cuff; gray oval denotes pillow; black arrow indicates movement direction.

Segmental spinal movements primarily by activating local spinal stabilizing muscles in the cervical, thoracic, and lumbar regions was assessed while supine or side-lying on an undeformable firm mattress, with the pressure cuff underneath participant’s body (Table 1, Fig 2). In order to ensure that each participant could selectively activate local spinal stabilizing muscles without using compensatory movements of other body parts, a physiotherapist who was certified and experienced in Nuerac^®^ method (Record^®^ AS) [31] instructed each participant how to correctly perform segmental spinal movements. Firstly, in order to obtain the feeling of correct movement, each participant lay comfortably on a mattress and was instructed to concentrate on and follow the small segmental spinal movements that the physiotherapist manually guided. Then each participant was asked to try to actively perform the segmental spinal movements with physiotherapist’s hands followed to monitor the correctness of movements and check unwanted muscle activities and movements of other body parts, such as postural shift. For example, when performing cervical spinal flexion movement, some participants might overtly activate sternocleidomastoid muscles. The physiotherapist then instructed the participants through verbal and tactile cues to help participants focus on activating deep cervical muscles (slight chin in using flexors longus capitis and longus colli) instead of using sternocleidomastoid muscles. Finally, physiotherapist checked if the movements could be performed correctly by each participant without cuing before the commencement of assessment.

Segmental spinal movements required each participant to actively and lightly contract segmental spinal stabilizing muscles against (or off) pressure cuff from baseline pressure to target pressure (increase or decrease pressure value from baseline by 10 mmHg). Baseline pressure and target pressure were determined by our pilot study and previous reports [29, 32]. Segmental spinal movements examined in this study were flexion in the cervical region (Table 1, Fig 2A); extension, side-shift, and rotation in the thoracic region (Table 1, Fig 2B–2D); and flexion, extension, side-shift, and rotation in the lumbar region (Table 1, Fig 2E–2H). The order of segmental spinal movements in the cervical, thoracic, and lumbar regions was random.

**Table 1:** Descriptions of segmental spinal movement assessment.

Regions	Movements and baseline/target pressure (mmHg)	Descriptions of test posture, location of cuff center, and test movement
**Cervical**		
	Flexion	Test posture: supine with pillows support under knees (approximately 130^o^ hip flexion)
	20 / 30 ± 0.5 mmHg	Cuff center: 4th vertebrae of cervical spine
		Test movement: slight chin in against pressure cuff with deep cervical flexor muscle contraction
**Thoracic**		
	Extension	Test posture: supine with pillows support under knees (approximately 130^o^ hip flexion)
	40 / 30 ± 0.5 mmHg	Cuff center: inferior angle of scapula
		Test movement: mid-trunk (thoracic spine) slightly away from pressure cuff
	Side-shift to right / left	Test posture: side-lying with both lower extremities flexed
	40 / 50 ± 0.5 mmHg	Cuff center: vertical line through right and left inferior angle of scapula
		Test movement: mid-trunk (thoracic spine) slightly downward push against pressure cuff
	Rotation to right / left	Test posture: supine with pillows support under knees (approximately 130^o^ hip flexion)
	40 / 50 ± 0.5 mmHg	Cuff center: inferior angle of scapula
		Test movement: right/left side of mid-trunk (thoracic spine) slightly downward push against pressure cuff
**Lumbar**		
	Flexion	Test posture: supine with pillows support under knees (approximately 130^o^ hip flexion)
	40 / 50 ± 0.5 mmHg	Cuff center: 3th vertebrae of the lumbar spine
		Test movement: lower trunk (lumbar spine) slightly downward push against pressure cuff
	Extension	Test posture: supine with pillows support under knees (approximately 130^o^ hip flexion)
	40 / 30 ± 0.5 mmHg	Cuff center: 3th vertebrae of lumbar spine
		Test movement: lower trunk (lumbar spine) slightly away from pressure cuff
	Side-shift to right / left	Test posture: side-lying with both lower extremities flexed
	40 / 50 ± 0.5 mmHg	Cuff center: vertical line through 3th vertebrae of lumbar spine
		Test movement: lower trunk (lumbar spine) slightly downward push against pressure cuff
	Rotation to right / left	Test posture: supine with pillows support under knees (approximately 130^o^ hip flexion)
	40 / 50 ± 0.5mmHg	Cuff center: 3th vertebrae of lumbar spine
		Test movement: right/left side of lower trunk (lumbar spine) slightly downward push against pressure cuff

For each segmental spinal movement, participants lay quietly in the test position and the examiner ensured the pressure value of pressure cuff was at baseline pressure for 2 minutes (Table 1). Participants were then instructed to perform the segmental spinal movement with mild muscle contraction (primarily by activating spinal stabilizing muscles) against (or off) the pressure cuff to achieve the target pressure (Table 1) while watching the pressure value displayed on the LCD screen of the microcontroller board. After participants were able to maintain at the target pressure for 5 seconds, they were then asked to sustain the muscle contraction without feedbacks from the LCD screen (turned off by the examiner) while maintaining the target pressure for 1 min (1-minute maintenance phase). To ensure the participants’ understanding of testing procedures, a trained physiotherapist demonstrated whole procedures and then each participant was instructed by the physiotherapist. Two to three practice trials were given before the actual test.

Pressure data of each segmental spinal movement during the 1-minute maintenance phase were measured and collected at a 50-Hz sampling rate by the pressure sensor and were then outputted to a personal computer for storage and subsequent analyses. Pressure variables examined in this study was the pressure deviation (defined as the root mean square of subtraction of target pressure from each pressure data point) for each segmental spinal movement during the 1-minute maintenance phase. Pressure deviation is viewed as the discrepancy between the given target pressure and the pressure derived from one’s contracting segmental stabilizing muscles of a spinal segment against (or off) the pressure cuff. A great pressure deviation (overshoot and undershoot the target pressure value) indicates one’s inferior ability to control and sustain segmental stabilizing muscle activities of a spinal segment against (or off) the pressure cuff in order to maintain at a given pressure level. Before the commencement of this study, a pilot investigation of between-day (within 5 days) reliability for all segmental spinal movements was conducted in 10 participants (5 with and 5 without AIS) and the findings revealed good to excellent reliability (intraclass correlation coefficients = 0.83–0.98 for all segmental spinal movements) of using the modified pressure biofeedback system to assess segmental spinal motor performance.

### Statistical analysis

Basic data, demographic characteristics, and pressure deviation for the AISG and CG are presented using descriptive analysis; these were compared between the groups using the Mann–Whitney test for continuous variables and chi-squared test for categorical variables. The significance level was set at a P value of < 0.05. All statistical analyses were performed using SPSS (version 22.0; SPSS Inc., Chicago, IL, USA).

## Results

### Participant characteristics

Twenty participants were included in the AISG, whereas 20 participants were in the CG. The groups were comparable for all demographic characteristics (Table 2). In the AISG, the average Cobb angle and angle of trunk rotation were respectively 22.1° ± 4.7° (range: 14°–35°) and 5.2° ± 2.6° (range: 2.5°–13.1°). Fourteen participants in the AISG had an S-curve type scoliosis, with seven having a major thoracic curve convex to the right and the rest having a major lumbar curve convex to the left. The remaining six participants in the AISG had a C-curve type scoliosis, with two having a major lumbar curve convex to the left and two each having a major thoracolumbar curve convex to the right and to the left.

**Table 2:** Participant’s characteristics of adolescent idiopathic scoliosis (AIS) and control groups.

Variables	AIS (n = 20)	Control (n = 20)	P value
**Female sex**	8 (40%)	7 (35%)	0.154
**Age (year)**	14.1 ± 2.3	13.6 ± 2.4	0.478
**Height (cm)**	159.2 ± 11.7	159.9 ± 11.1	0.883
**Weight (kg)**	47.9 ± 10.6	55.5 ± 13.6	0.121
**Body mass index (kg/m**^**2**^**)**	18.7 ± 2.3	21.7 ± 4.7	0.055
**Cobb angle (degree)**	22.1 ± 4.7	-	-
**Angle of trunk rotation (degree)**	5.2 ± 2.6	-	-
**Type of scoliosis**			
** S curve**			
**Thoracic scoliosis**			
** Convex to right**	7 (35%)	-	-
**Lumbar scoliosis**			
** Convex to left**	7 (35%)	-	-
** C curve**			
** Thoracolumbar scoliosis**			
** Convex to right**	2 (10%)	-	-
** Convex to left**	2 (10%)	-	-
** Lumbar scoliosis**			
** Convex to left**	2 (10%)	-	-

Data are presented as mean ± standard deviation or number (percentage).

### Comparison of pressure deviation

Table 3 lists the pressure deviations of all segmental spinal movements for the AISG and CG. The pressure deviation for cervical flexion significantly differed between the AISG and CG, with the AISG exhibiting a greater deviation from the target pressure.

**Table 3:** Pressure deviation of segmental spinal movements for cervical, thoracic, and lumbar regions.

Movements	AIS (n = 20)	Control (n = 20)	Mann-Whitney U	P value
**Cervical**				
** Flexion**	2.98 ± 1.27	1.30 ± 0.98	51	<0.001*
**Thoracic**				
** Extension**	2.22 ± 2.62	1.57 ± 1.07	112	0.017*
** Side-shift to right**	2.53 ± 1.73	1.41 ± 1.22	102	0.008*
** Side-shift to left**	2.18 ± 1.92	1.90 ± 0.97	124	0.040*
** Rotation to right**	2.89 ± 2.26	1.29 ± 0.49	60	<0.001*
** Rotation to left**	2.51 ± 3.15	1.60 ± 1.52	125	0.042*
**Lumbar**				
** Flexion**	2.90 ± 1.52	1.51 ± 1.59	89	0.003*
** Extension**	1.97 ± 1.56	1.21 ± 0.86	116	0.023*
** Side-shift to right**	4.48 ± 4.07	1.60 ± 1.23	94	0.004*
** Side-shift to left**	3.13 ± 3.46	2.00 ± 1.25	78	0.001*
** Rotation to right**	2.40 ± 2.29	1.35 ± 0.91	90	0.003*
** Rotation to left**	2.41 ± 4.08	1.41 ± 1.08	95	0.005*

*P < 0.05. Data are presented as median ± inter-quartile range (unit: mmHg).

The AISG demonstrated a significantly greater pressure deviations for thoracic extension as well as for thoracic side-shift movements to the right and to the left than did the CG. In addition, the pressure deviations for thoracic rotation to the right and to the left significantly differed between the AISG and CG, with the AISG exhibiting greater deviations from the target pressures (Table 3).

The AISG demonstrated significantly greater pressure deviations for lumbar flexion and extension than did the CG. Furthermore, the pressure deviations for lumbar side-shift movements to the right and to the left were higher in the AISG than in the CG. The pressure deviations of lumbar rotation to the right and to the left were higher in the AISG than in the CG (Table 3).

## Discussion

The results of this study indicate that the modified pressure biofeedback unit was feasible for assessing and sensitive to alterations on the performance of segmental spinal movement control in AIS. Notably, the segmental spinal movement control of the cervical, thoracic, and lumbar regions was all affected in AISG, but the between-group differences in terms of pressure values were not large. Furthermore, we determined that the pressure deviation for all examined segmental spinal movements were greater in the AISG. This finding thus supports the hypothesis that the movement control of the axial motor system is affected in AIS, as indicated by a poorer ability of AISG to grade and sustain steady force output of segmental spinal stabilizing muscles when compared with CG. The significant minor impairments may compromise the ability of individuals with AIS to adapt and to maintain normal spinal curvature during the rapid growth period, thus individuals with AIS exhibit progressive deterioration of spinal curvature during this period.

The maneuvers used in this study and the pre-test physiotherapist’s instructions and practice sessions secured the participants to focus on segmental spinal movement control rather than using compensation of other body parts and ensured the participants to use somatosensory information of trunk to actively scale and sustain muscle activity without external feedback to fulfil the requirements of movement tasks in this study. We speculate that the poor performance on segmental spinal movements in AISG may be related to impaired proprioception of the trunk, dysfunction of the central sensoriomotor integration of the axial motor system, and morphological changes in the trunk muscles.

To our knowledge, no previous study has examined truncal proprioceptive function in AIS. The test maneuvers used in this study provided a window for investigating truncal proprioceptive function. Our findings of a greater pressure deviation of segmental spinal movements together with previous reports that a larger error for a joint repositioning test as well as a higher joint movement perception threshold of the upper extremities [33] and lower extremities [10] suggested impaired proprioception in AIS should be a systematic problem. Proprioceptive deficits of the trunk in AIS may impeding the AISG from appropriately fine-tuning segmental stabilizing muscle activities by using afferent information from the adjacent spinal segments to meet the task demands. Consequently, the AISG demonstrated a poorer performance on segmental spinal movement control than the CG. These findings indicate that body awareness training is an important component of rehabilitation management of AIS [34, 35].

The poor segmental spinal movement control in AIS observed in this study may also be associated with dysfunctional central sensorimotor control [10], altered sensory weighting/reweighting mechanism [14, 15], or abnormal interhemispheric asymmetric activation [13] that were proposed in previous studies. Aberrant central sensorimotor processing and control in AIS jeopardize the using somatosensory information form the concave side and convex side of the trunk to scale activities of segmental spinal stabilizing muscles. Our findings systematically demonstrate that compared with the CG, the AISG exhibited greater pressure deviations for all segmental spinal movements involving the active sustained contraction of segmental stabilizing muscles around cervical, thoracic, and lumbar regions. Examination of the associations of brain activity, somatosensation of trunk, and muscle activities of trunk may provide better insight into the problem.

Asymmetric muscle thickness in the deep thoracic paraspinal muscles [18], lumbar multifidus [18, 19], obliquus internus and externus [20], and transverse abdominus [21], as well as with type I muscle atrophy of the paraspinal muscles [36] may be associated with our observation that AISG had poor local spinal movement control. Such morphological alterations in muscles (asymmetry and biomechanical disadvantage) may require differential activation of segmental stabilizing muscles on both sides that may impose a greater demand on axial motor control system; therefore, negatively influence the motor performance. The associations between asymmetric muscle thickness of truncal muscles and poor local axial motor control need to be examined in future study using sonography and electromyography.

Our findings provide clues for clinical assessment and management of AIS. According to functional roles, trunk muscles can be classified as stabilizers and mobilizers [37]. The former is primarily responsible for stability and physiological alignment of spine and the latter is responsible for a range of trunk movements. The scoliotic spine can be interpreted as that the stability and physiological alignment of spine are no longer maintained by elemental components, particularly the trunk stabilizing muscles. Therefore, the maneuvers and assessment method used in this study allow examination of specific function of trunk stabilizers and inform which spinal segments and movement directions are affected as well as the extent of impairments. The information is important for physiotherapeutic management AIS. Our findings also suggest that training of trunk stabilizers should be incorporated into rehabilitation program for AIS. This proposition is supported by previous study reporting that the effect of adding on training of trunk stabilizing muscles was superior to traditional exercise in reducing scoliotic curve and pain in AIS [38]. Furthermore, we propose that training of trunk stabilizers should be in all three planes and be introduced in the beginning of rehabilitation program in order to amend impairments of segmental spinal stabilizing muscles and establish a stable spine, and then progresses from static to dynamic corrective exercise to enhance functional integration of trunk stabilizer and mobilizer to restore a symmetric, neutral spine.

This research has several limitations. First, we examined axial movement control in supine and side-lying positions; whether similar findings might be obtained in upright positions (i.e., sitting or standing) remains unknown. Second, our sample size was small, and the AIS group encompassed different types of scoliosis of mild to moderate severity; therefore, a subgroup analysis could not be performed. Whether impaired axial movement control is associated with AIS types and severity warrants further investigation. Third, the maneuvers employed in this study to activate segmental spinal stabilizers are according to systematically developed clinical approach (i.e., Nuerac^®^ method (Record^®^ AS)) [31] and the using pressure biofeedback to monitor segmental spinal movements has been reported in several studies [27, 28, 30]. However, further study is required to monitor activities of these muscles and kinematics of body movements while performing the pressure biofeedback assessment in AIS.

## Conclusion

The modified pressure biofeedback unit was feasible for assessing and sensitive to alterations on the performance of segmental spinal movement control in AIS. AISG exhibited a poorer ability to grade and sustain muscle activity in the axial regions, suggesting training of segmental spinal movement control on frontal, sagittal, and transverse planes were required.

## References

[1] Cobb JR. Outline for the study of scoliosis. Am Acad Othop Surg Inst Course Lect. 1948;5:261–75.

[2] Konieczny MR, Senyurt H, Krauspe R. Epidemiology of adolescent idiopathic scoliosis. J Child Orthop. 2013;7(1):3–9. doi: 10.1007/s11832-012-0457-4 24432052 10.1007/s11832-012-0457-4 PMC3566258

[3] Weinstein SL, Ponseti IV. Curve progression in idiopathic scoliosis. J Bone Joint Surg Am. 1983;65(4):447–55. 6833318

[4] Weinstein SL, Dolan LA, Cheng JC, Danielsson A, Morcuende JA. Adolescent idiopathic scoliosis. Lancet. 2008;371(9623):1527–37. doi: 10.1016/S0140-6736(08)60658-3 18456103 10.1016/S0140-6736(08)60658-3

[5] Goldberg MS, Mayo NE, Poitras B, Scott S, Hanley J. The Ste-Justine Adolescent Idiopathic Scoliosis Cohort Study. Part II: Perception of health, self and body image, and participation in physical activities. Spine (Phila Pa 1976). 1994;19(14):1562–72. 7939992

[6] Asher MA, Burton DC. Adolescent idiopathic scoliosis: natural history and long term treatment effects. Scoliosis. 2006;1(1):2 doi: 10.1186/1748-7161-1-2 16759428 10.1186/1748-7161-1-2 PMC1475645

[7] Wang WJ, Yeung HY, Chu WC, Tang NL, Lee KM, Qiu Y, et al Top theories for the etiopathogenesis of adolescent idiopathic scoliosis. J Pediatr Orthop. 2011;31(1 Suppl):S14–27. doi: 10.1097/BPO.0b013e3181f73c12 21173615 10.1097/BPO.0b013e3181f73c12

[8] Burwell RG, Clark EM, Dangerfield PH, Moulton A. Adolescent idiopathic scoliosis (AIS): a multifactorial cascade concept for pathogenesis and embryonic origin. Scoliosis Spinal Disord. 2016;11:8 doi: 10.1186/s13013-016-0063-1 27252984 10.1186/s13013-016-0063-1 PMC4888516

[9] Hefti F. Pathogenesis and biomechanics of adolescent idiopathic scoliosis (AIS). J Child Orthop. 2013;7(1):17–24. doi: 10.1007/s11832-012-0460-9 24432054 10.1007/s11832-012-0460-9 PMC3566249

[10] Pialasse JP, Mercier P, Descarreaux M, Simoneau M. Sensorimotor Control Impairment in Young Adults With Idiopathic Scoliosis Compared With Healthy Controls. J Manipulative Physiol Ther. 2016;39(7):473–479. doi: 10.1016/j.jmpt.2016.06.001 27544925 10.1016/j.jmpt.2016.06.001

[11] Lombardi G, Akoume MY, Colombini A, Moreau A, Banfi G. Biochemistry of adolescent idiopathic scoliosis. Adv Clin Chem. 2011;54:165–182. 21874761 10.1016/b978-0-12-387025-4.00007-8

[12] Herman R, Mixon J, Fisher A, Maulucci R, Stuyck J. Idiopathic scoliosis and the central nervous system: a motor control problem. The Harrington lecture, 1983. Scoliosis Research Society. Spine. 1985;10(1):1–14. 3885413 10.1097/00007632-198501000-00001

[13] Domenech J, Garcia-Marti G, Marti-Bonmati L, Barrios C, Tormos JM, Pascual-Leone A. Abnormal activation of the motor cortical network in idiopathic scoliosis demonstrated by functional MRI. Eur Spine J. 2011;20(7):1069–78. doi: 10.1007/s00586-011-1776-8 21499781 10.1007/s00586-011-1776-8 PMC3176702

[14] Pialasse JP, Descarreaux M, Mercier P, Simoneau M. Sensory reweighting is altered in adolescent patients with scoliosis: Evidence from a neuromechanical model. Gait Posture. 2015;42(4):558–563. doi: 10.1016/j.gaitpost.2015.08.013 26371828 10.1016/j.gaitpost.2015.08.013

[15] Simoneau M, Mercier P, Blouin J, Allard P, Teasdale N. Altered sensory-weighting mechanisms is observed in adolescents with idiopathic scoliosis. BMC Neurosci. 2006;7:68 doi: 10.1186/1471-2202-7-68 17052338 10.1186/1471-2202-7-68 PMC1633738

[16] Cheung J, Halbertsma JP, Veldhuizen AG, Sluiter WJ, Maurits NM, Cool JC, et al A preliminary study on electromyographic analysis of the paraspinal musculature in idiopathic scoliosis. Eur Spine J. 2005;4(2):130–7. 10.1007/s00586-004-0780-7 PMC3476698 15368104

[17] Chwala W, Koziana A, Kasperczyk T, Walaszek R, Płaszewski M. Electromyographic assessment of functional symmetry of paraspinal muscles during static exercises in adolescents with idiopathic scoliosis. Biomed Res Int. 2014;2014:573276 doi: 10.1155/2014/573276 25258713 10.1155/2014/573276 PMC4167233

[18] Zapata KA, Wang-Price SS, Sucato DJ, Dempsey-Robertson M. Ultrasonographic measurements of paraspinal muscle thickness in adolescent idiopathic scoliosis: a comparison and reliability study. Pediatr Phys Ther. 2015;27(2):119–25. doi: 10.1097/PEP.0000000000000131 25695194 10.1097/PEP.0000000000000131

[19] Kennelly KP, Stokes MJ. Pattern of asymmetry of paraspinal muscle size in adolescent idiopathic scoliosis examined by real-time ultrasound imaging. A preliminary study. Spine. 1993;18(7):913–7. 8316893 10.1097/00007632-199306000-00017

[20] Yang HS, Yoo JW, Lee BA, Choi CK, You JH. Inter-tester and intra-tester reliability of ultrasound imaging measurements of abdominal muscles in adolescents with and without idiopathic scoliosis: a case-controlled study. Biomed Mater Eng. 2014;24(1):453–458. doi: 10.3233/BME-130830 24211927 10.3233/BME-130830

[21] Linek P, Saulicz E, Wolny T, Mysliwiec A, Gogola A. Ultrasound evaluation of the symmetry of abdominal muscles in mild adolescent idiopathic scoliosis. J Phys Ther Sci. 2015;27(2):465–468. doi: 10.1589/jpts.27.465 25729192 10.1589/jpts.27.465 PMC4339162

[22] Panjabi MM. The stabilizing system of the spine. Part I. Function, dysfunction, adaptation, and enhancement. J Spinal Disord. 1992;5(4):383–9. 1490034 10.1097/00002517-199212000-00001

[23] Panjabi MM. The stabilizing system of the spine. Part II. Neutral zone and instability hypothesis. J Spinal Disord. 1992;5(4):390–6. 1490035 10.1097/00002517-199212000-00002

[24] Akuthota V, Ferreiro A, Moore T, Fredericson M. Core stability exercise principles. Curr Sports Med Rep. 2008;7(1):39–44. doi: 10.1097/01.CSMR.0000308663.13278.69 18296944 10.1097/01.CSMR.0000308663.13278.69

[25] Shirado O, Ito T, Kaneda K, Strax TE. Kinesiologic analysis of dynamic side-shift in patients with idiopathic scoliosis. Arch Phys Med Rehabil. 1995;76(7):621–6. 7605180 10.1016/s0003-9993(95)80630-x

[26] Guyot MA, Agnani O, Peyrodie L, Samantha D, Donze C, Catanzariti JF. Cervicocephalic relocation test to evaluate cervical proprioception in adolescent idiopathic scoliosis. Eur Spine J. 2016;25(10):3130–3136. doi: 10.1007/s00586-016-4551-z 27072549 10.1007/s00586-016-4551-z

[27] Carlsson H, Rasmussen-Barr E. Clinical screening tests for assessing movement control in non-specific low-back pain. A systematic review of intra- and inter-observer reliability studies. Man Ther. 2013;18(2):103–110. doi: 10.1016/j.math.2012.08.004 23018080 10.1016/j.math.2012.08.004

[28] Hagins M, Adler K, Cash M, Daugherty J, Mitrani G. Effects of practice on the ability to perform lumbar stabilization exercises. J Orthop Sports Phys Ther. 1999;29(9):546–55. doi: 10.2519/jospt.1999.29.9.546 10518297 10.2519/jospt.1999.29.9.546

[29] Chattanooga Group Inc. Stabilizer Pressure Bio-feedback. Operating Insructions. Hixson: Chattanooga Group, Inc.; 2005.

[30] Iqbal ZA, Rajan R, Khan SA, Alghadir AH. Effect of deep cervical flexor muscles training using pressure biofeedback on pain and disability of school teachers with neck pain. J Phys Ther Sci. 2013;25(6):657–61. doi: 10.1589/jpts.25.657 24259822 10.1589/jpts.25.657 PMC3805007

[31] Kirkesola G. The neurac method. J Fysioterapeuten. 2009;76:16–25.

[32] Hudswell S, von Mengersen M, Lucas N. The cranio-cervical flexion test using pressure biofeedback: A useful measure of cervical dysfunction in the clinical setting? Int J Osteopath Med. 2005;8:98–105.

[33] Cook SD, Harding AF, Burke SW, Whitecloud TS, Barrack RL, Leinhardt TM. Upper extremity proprioception in idiopathic scoliosis. Clin Orthop Relat Res. 1986(213):118–24. 3780080

[34] Romano M, Negrini A, Parzini S, Tavernaro M, Zaina F, Donzelli S, et al SEAS (Scientific Exercises Approach to Scoliosis): a modern and effective evidence based approach to physiotherapic specific scoliosis exercises. Scoliosis. 2015;10:3 doi: 10.1186/s13013-014-0027-2 25729406 10.1186/s13013-014-0027-2 PMC4344739

[35] Weiss HR, Moramarco MM, Borysov M, Ng SY, Lee SG, Nan X, et al Postural Rehabilitation for Adolescent Idiopathic Scoliosis during Growth. Asian Spine J. 2016;10(3):570–81. doi: 10.4184/asj.2016.10.3.570 27340540 10.4184/asj.2016.10.3.570 PMC4917779

[36] Slager UT, Hsu JD. Morphometry and pathology of the paraspinous muscles in idiopathic scoliosis. Dev Med Child Neurol. 1986;28(6):749–756. 3817313 10.1111/j.1469-8749.1986.tb03928.x

[37] Willson JD, Dougherty CP, Ireland ML, Davis IM. Core stability and its relationship to lower extremity function and injury. J Am Acad Orthop Surg. 2005;13(5):316–25. 16148357 10.5435/00124635-200509000-00005

[38] Gur G, Ayhan C, Yakut Y. The effectiveness of core stabilization exercise in adolescent idiopathic scoliosis: A randomized controlled trial. Prosthet Orthot Int. 2017;41(1):1–8. 10.1177/0309364616664151 27625122

